# Delayed-type hypersensitivity (DTH) test anergy does not impact CD4 reconstitution or normalization of DTH responses during antiretroviral therapy

**DOI:** 10.7448/IAS.17.1.18799

**Published:** 2014-02-04

**Authors:** Natascha M Minidis, Octavio Mesner, Brian K Agan, Jason F Okulicz

**Affiliations:** 1 Infectious Disease Clinical Research Program, Uniformed Services University of the Health Sciences Bethesda, MD, USA; 2 Infectious Disease Service, San Antonio Military Medical Center San Antonio, TX, USA

**Keywords:** HIV, HAART, antiretroviral therapy, delayed-type hypersensitivity, DTH, CD4 cell count, anergy, anergic

## Abstract

**Introduction:**

Delayed-type hypersensitivity (DTH) testing is an *in vivo* assessment of cell-mediated immunity. Although highly active antiretroviral therapy (HAART) improves immunologic parameters, the relationship between DTH responsiveness and CD4 gains on HAART is not completely understood. We investigated CD4 reconstitution and the change in DTH responses from treatment baseline through 24 months of viral load (VL)-suppressive HAART in the U.S. Military HIV Natural History Study.

**Methods:**

Treatment-naïve subjects with VL <400 copies/mL after ≥24 months on HAART were included (*n*=302). DTH testing consisted of ≥3 recall antigens, and responses were classified by the number of positive skin tests: anergic (0–1) or non-anergic (≥2). Pre-HAART DTH results were compared for the outcome of CD4 reconstitution at 24 months of HAART. Improvement in DTH responses was also analyzed for those anergic before HAART initiation.

**Results:**

Non-anergic responses were observed in 216 (72%) participants, while 86 (28%) individuals were anergic prior to HAART initiation. Demographically there were similar distributions of age at HIV diagnosis and HAART initiation, as well as gender and race or ethnicity. There were no significant differences between non-anergic and anergic participants in pre-HAART CD4 count (409 cells/μL, interquartile range (IQR) 315–517 vs. 373 cells/μL, IQR 228–487; *p*=0.104) and VL (4.3 log_10_ copies/mL, IQR 3.4–4.9 vs. 4.4 log_10_ copies/mL, IQR 3.6–5.0; *p*=0.292). Median CD4 gains 24 months after HAART initiation were similar between the non-anergic (220 cells/μL, IQR 115–358) and anergic groups (246 cells/μL, IQR 136–358; *p*=0.498). For individuals anergic before HAART initiation, DTH normalization occurred at 24 months post-HAART in the majority of participants (51 of 86, 59%). Normalization of DTH responses was not associated with CD4 count at HAART initiation (OR 0.73, 95% CI 0.47, 1.09 per 100 cells; *p*=0.129) nor with AIDS diagnoses prior to HAART (OR 0.34, 95% CI 0.04, 2.51; *p*=0.283).

**Conclusions:**

DTH responsiveness has been shown to predict HIV disease progression independent of CD4 count in untreated individuals. In the setting of HAART, pre-HAART anergy does not appear to impact CD4 gains or the ability to normalize DTH responses after 24 months of VL-suppressive HAART.

## Introduction

With the increased success of newer highly active antiretroviral therapy (HAART) regimens, the morbidity and mortality of patients taking HAART in the United States continue to decline [1–4]. The goals of HIV treatment include improvement in clinical signs and symptoms of HIV disease, prevention of HIV transmission, suppression of plasma viral load (VL), and improvement in immunologic function. In clinical practice, the immunologic outcome of HAART is typically measured by CD4 cell reconstitution. However, additional information about a patient's immunologic health can be ascertained by evaluating for AIDS outcomes and performing other laboratory studies, including serologic responses to vaccines. Another method of assessing immune function that can be readily performed in clinical settings is delayed-type hypersensitivity (DTH) testing. As an *in vivo* assessment of cell-mediated immunity (CMI), DTH testing is performed by intradermal application of antigens with subsequent measurement of the local cutaneous reaction.

DTH responsiveness is typically reduced in patients living with HIV compared to seronegative individuals [5, 6]. For example, >90% of HIV-uninfected individuals will be reactive, or non-anergic, to DTH recall antigens [7, 8]. In contrast, approximately 60% of those living with HIV have non-anergic DTH responses [9, 10]. In individuals with untreated HIV, DTH responsiveness is typically greater in those with CD4 cell counts >400 cells/μL compared to those with <400 cells/μL [6], with anergy to DTH antigens often observed in those with very low CD4 counts [5, 11, 12]. DTH responses have also been shown to decline in parallel with CD4 cells in untreated patients [10, 11]. Despite the association between DTH and magnitude of CD4 count, DTH responsiveness has been shown to be a predictor of HIV disease progression independent of CD4 cell count in the absence of HAART [5, 6, 8, 9].

DTH responses have also been studied in patients receiving HAART. In addition to immune reconstitution measured clinically by CD4 cell gains, several studies demonstrated an improvement in DTH responses after initiation of HAART [12–14]. However, despite successful treatment, DTH responsiveness remains impaired for many patients on HAART [12, 15]. Since DTH responses have been used to prognosticate HIV outcomes in untreated individuals, DTH testing may have potential for use as a predictor for HAART outcomes. In order to investigate the predictive value of DTH testing in the current study, our first aim was to retrospectively analyse whether non-anergic pre-HAART DTH status predicted more robust CD4 cell gains after initiation of VL-suppressive HAART in the U.S. Military HIV Natural History Study (NHS). For our second aim, we studied the frequency of post-HAART DTH normalization in those with anergy prior to HAART initiation (HI) and the factors associated with improved DTH responses.

## Methods

The NHS is a prospective observational cohort of over 5500 military members, dependents, and beneficiaries who have been evaluated for HIV-1 positive status in the military healthcare system since 1986 [16]. Study visits occur approximately every 6 months at selected US military treatment facilities, with systematic data collection including demographic characteristics, laboratory and treatment records, and reports of clinical events with medical record confirmation. All participants enrolled in this Institutional Review Board-approved study provided written informed consent and were ≥18 years of age.

The NHS database was queried for participants on HAART who also received serial DTH testing for this retrospective analysis. Participants achieving a VL <400 copies/mL within six months of their initial HAART regimen and maintaining VL suppression for ≥24 months were included. The analysis was restricted to those achieving VL suppression because the outcome of immune reconstitution during HAART was the primary focus of this study and maximal CD4 gains occur when VL is fully suppressed. The association between pre-HAART DTH response and the outcome of VL suppression was not analyzed because data for some confounders, including HIV genotype and HAART adherence, were not available. Individuals were required to have DTH testing within six months prior to HI and repeat DTH testing performed at 24 months on HAART. DTH testing was performed according to standardized protocols, as previously described [5, 6, 8, 13]. A total of 0.1 mL of each antigen was applied to the forearm intradermally according to the Mantoux method, and a positive test was defined as ≥5 mm of induration after 48 h. The most recent antigens and concentrations included tetanus toxoid (Lederle, 1.6 Lf/mL; 1:100 dilution), mumps (Connaught, 40 CFU/mL), trichophyton (Holister-Stier, 1:500 dilution), and candida (Walter Reed Army Institute of Research, 200 PNU/mL). Participants received a panel of 3–4 antigens, with the majority receiving three antigens because trichophyton was removed from the market in 1996. DTH responses were categorized by the number of positive skin tests: anergic (0–1) or non-anergic (≥2), as previously described [5].

Baseline characteristic *p*-values were calculated using *t*-tests or Wilcoxon tests for continuous variables depending on normality, and chi-squared tests or Fisher's exact tests for categorical variables depending on sparse cells. To estimate the impact of DTH status at HI on post-HAART immune function, we modelled CD4 gains at 24 months after HI with multiple linear regression while adjusting for other relevant covariates. Multiple logistic regression was used to understand factors associated with post-HAART DTH normalization among those who were anergic at HI.

## Results

A total of 302 participants met inclusion criteria, of which 216 (72%) were non-anergic and 86 (28%) were anergic by DTH testing prior to HI (Table 1). Demographic characteristics, including age at HIV diagnosis and gender, were similar between groups, although European Americans were more likely to be anergic compared to those of other races or ethnicities (*p*=0.015). Median CD4 count at diagnosis was significantly higher in the non-anergic group (497 cells/μL, interquartile range (IQR) 360–656) compared to the anergic group (432 cells/μL, IQR 279–540; *p*=0.014); however, VL was no different (4.5 log_10_ copies/mL, IQR 4.0–4.9 vs. 4.6 log_10_ copies/mL, IQR 4.0–5.1; *p*=0.335).

**Table 1:** Baseline characteristics at HAART initiation

		Pre-HAART DTH test results		
			
	All participants	Anergic	Non-anergic	*p*
Participants, *n*	302	86	216	
Age at HIV diagnosis (years)	31 (26, 36)	32 (27, 38)	30 (26, 36)	0.103
Gender				0.908
Male	280 (93%)	80 (93%)	200 (93)	
Female	22 (7%)	6 (7%)	16 (7%)	
Race or ethnicity				0.015
European-American	169 (56%)	57 (66%)	112 (52%)	
African-American	97 (32%)	17 (20%)	80 (37%)	
Hispanic/other	36 (12%)	12 (14%)	24 (11%)	
Year of HIV diagnosis	1995 (1991, 1998)	1995 (1991, 1998)	1995 (1991, 1998)	0.595
CD4 count at HIV diagnosis (cells/μL)	488 (338, 637)	432 (279, 540)	497 (360, 656)	0.014
VL at HIV diagnosis (log_10_ copies/mL)	4.5 (4, 5)	4.6 (4, 5.1)	4.5 (4, 4.9)	0.335
Year of HAART initiation	1998 (1997, 1999)	1998 (1996, 1999)	1998 (1997, 1999)	0.785
Age at HAART initiation (years)	35 (31, 40)	35 (32, 41)	35 (30, 39)	0.242
AIDS prior to HAART				0.015
No	287 (95%)	77 (90%)	210 (97%)	
Yes	15 (5%)	9 (10%)	6 (3%)	
CD4 count at HAART initiation (cells/μL)	404 (287, 510)	373 (228, 487)	409 (315, 517)	0.104
VL at HAART initiation (log_10_ copies/mL)	4.3 (3.5, 4.9)	4.4 (3.6, 5)	4.3 (3.4, 4.9)	0.292
Initial HAART regimen				0.062
PI based	205 (68%)	67 (78%)	138 (64%)	
NNRTI based	73 (24%)	14 (16%)	59 (27%)	
Other	24 (8%)	5 (6%)	19 (9%)	

Results expressed as a number and percentage or as a median and interquartile range. DTH, delayed-type hypersensitivity; HAART, highly active antiretroviral therapy; VL, viral load; PI, protease inhibitor; NNRTI, non-nucleoside reverse transcriptase inhibitor.

The year of HI was typically in the early HAART era (1998 for both groups). The median CD4 at HI was higher for non-anergic (409 cells/μL, IQR 315–517) versus anergic (373 cells/μL, IQR 228–487) participants, but this was not statistically significant (*p*=0.104). VL at HI was also similar between groups. AIDS diagnoses prior to HI were more common in the anergic group (*n*=9, 10%) compared to the non-anergic group (*n*=6, 3%; *p*=0.015). The initial HAART regimen most commonly used was protease inhibitor (PI) based (*n*=205, 68%) compared to non-nucleoside reverse transcriptase inhibitor (NNRTI) based (*n*=73, 24%) and other regimens that typically contained both PIs and NNRTIs (*n*=24, 8%). For DTH test outcomes, there was a trend toward greater PI-based regimen use in the anergic group (*n*=67, 78%) compared to the non-anergic group (*n*=138, 64%; *p*=0.062).

**Figure 1:**
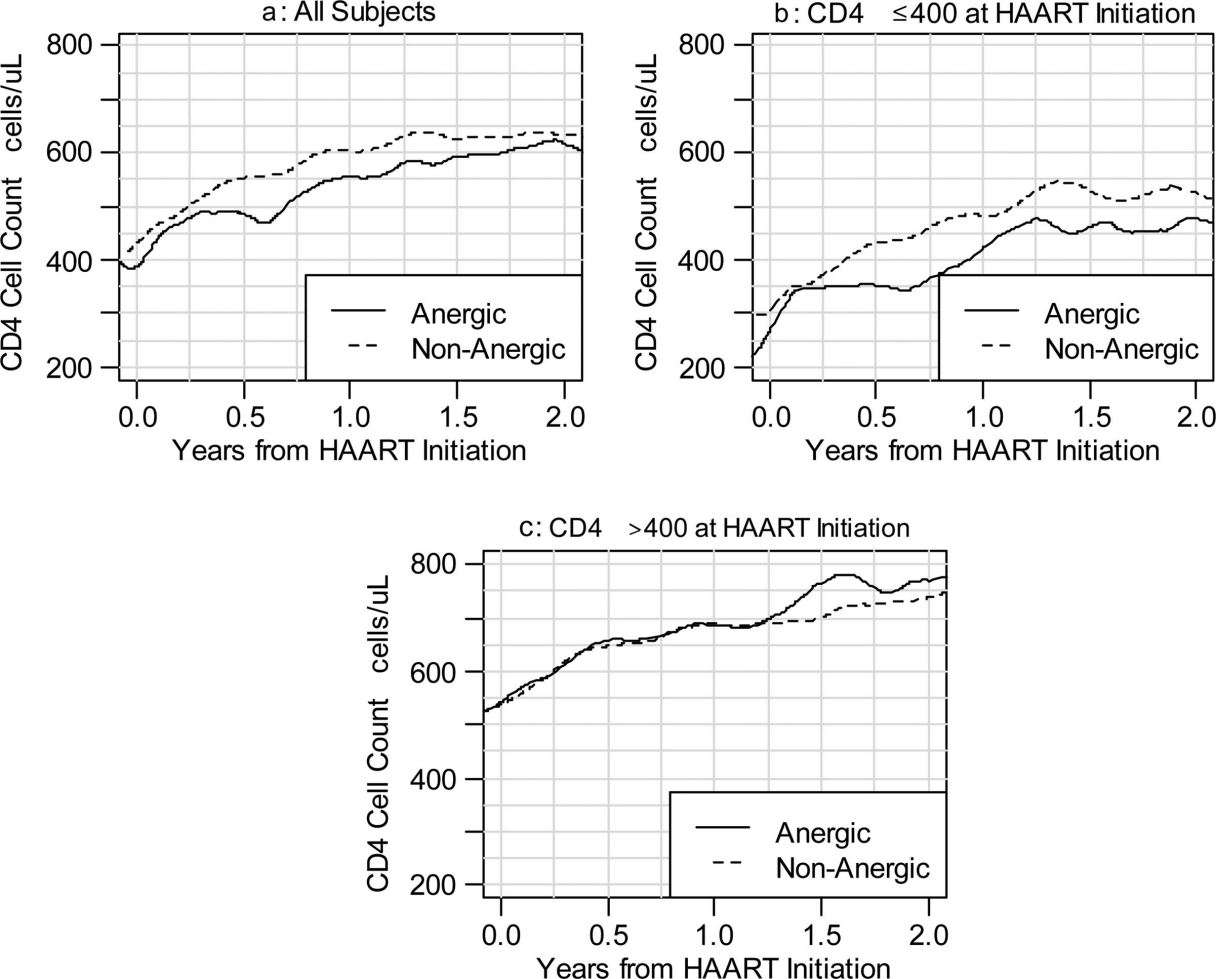
Post-HAART CD4 gains by non-anergic versus anergic pre-HAART DTH response for (a) all participants and stratified by (b) CD4 count ≤400 cells/μL or (c) >400 cells/μL at HAART initiation.

The median CD4 cell gains after HI were similar for non-anergic compared to anergic subjects, even when stratified by pre-HAART CD4 count ≤400 or >400 cells/μL (Figure 1). At 24 months of HAART, non-anergic participants gained a median 220 cells/μL (IQR 115–358) versus 246 cells/μL (IQR 136–358) for anergic participants (*p*=0.498). Linear regression was performed to investigate the factors associated with CD4 gains (Table 2). Non-anergic participants at HI had smaller, non-significant CD4 gains than those who were anergic after adjusting for CD4 at HI, race or ethnicity, gender, initial HAART regimen, and prior AIDS diagnosis. For every additional 100 CD4 cells/μL at HI, overall CD4 gains at 24 months were reduced (−23.8 cells/μL, 95% CI: −38.38 to −9.23; *p*<0.001). Other variables, including diagnosis of AIDS prior to HI or type of HAART regimen, were not significantly associated with CD4 gains at 24 months.

**Table 2:** Regression analyses for adjusted CD4 gains and DTH normalization at 24 months of antiretroviral therapy

	Factors associated with CD4 gain		Factors associated with DTH normalization	
		
Characteristic	OR (95% CI)	*p*	OR (95% CI)	*p*
Pre-HAART DTH result anergic	0	(ref)	–	–
Pre-HAART DTH result non-anergic	−38.07 (−95.55, 19.41)	0.193	–	–
Gender				
Male	0	(ref)	–	–
Female	−70.84 (−162.3, 20.62)	0.128	1.68 (0.15, 42.49)	0.694
Race				
European-American	0	(ref)	–	–
African-American	40.46 (−13.98, 94.91)	0.145	17.23 (2.53, 371.19)	0.015
Hispanic/other	21.94 (−57.39, 101.27)	0.587	7.95 (1.13, 93.34)	0.060
AIDS prior to HAART initiation				
No	0	(ref)	–	–
Yes	−47.04 (−159.06, 64.98)	0.409	0.34 (0.04, 2.51)	0.283
Age at HAART initiation (years)	−22.46 (−53.4, 8.48)	0.154	1.62 (0.74, 3.76)	0.240
CD4 count at HAART initiation (per 100 cells/μL)	−23.8 (−38.38, −9.23)	0.001	0.73 (0.47, 1.09)	0.129
Initial HAART regimen				
PI based	0	(ref)	–	–
NNRTI based	4.21 (−53.28, 61.71)	0.885	0.2 (0.04, 0.82)	0.030
Other	−21.7 (−110.6, 67.21)	0.631	0.44 (0.04, 4.97)	0.490

CD4 gains analyzed by linear regression; DTH normalization analyzed by logistic regression. DTH, delayed-type hypersensitivity; HAART, highly active antiretroviral therapy; PI, protease inhibitor; NNRTI, non-nucleoside reverse transcriptase inhibitor.

The subset of participants who were anergic at HI (*n*=86) was studied for factors associated with normalization of DTH responses at 24 months of HAART (Table 2). Of these, 51 (59%) normalized DTH responses from anergic to non-anergic. Interestingly, logistic regression showed that neither CD4 cell count (OR 0.73, 95% CI: 0.47–1.09 per 100 cells; *p*=0.129) nor AIDS prior to HI (OR 0.34, 95% CI: 0.04–2.51; *p*=0.283) was associated, with DTH normalization. Race or ethnicity was associated with DTH status at 24 months with African Americans (OR 17.23, 95% CI 2.53, 371.19; *p*=0.015) and Hispanic/others (OR 7.95 95% CI: 1.13–93.34; *p*=0.06) more likely to become non-anergic than European Americans. Those whose first HAART regimen was NNRTI based compared to PI based were less likely to become non-anergic (OR 0.2, 95% CI: 0.04–0.82; *p*=0.03), but no other covariates were associated with DTH normalization.

## Discussion

We investigated whether pre-HAART DTH responses predicted CD4 gains 24 months after initiation of VL-suppressive HAART. Although CD4 reconstitution was robust in both groups, DTH responsiveness before HAART did not predict CD4 gains during treatment. Effective HAART results in increased CD4 counts and reduced risk of opportunistic infections, but HAART does not fully restore immune function [17–19]. For those anergic prior to HAART in our study, most participants, but not all, became non-anergic after 24 months of HAART. Interestingly, the ability to normalize DTH responses was not associated with pre-HAART CD4 count or prior diagnosis of AIDS. Thus, anergic DTH responses do not appear to impact the ability to reconstitute CD4 cells or reverse cutaneous anergy to recall antigens after HI.

The utility of DTH testing has been demonstrated in many studies and was previously recommended in the evaluation and disease staging of patients living with HIV. For example, DTH responsiveness has been shown to be an independent predictor of opportunistic infections and death [5, 6, 8, 9]. The US Centers for Disease Control (CDC) previously recommended testing for anergy in conjunction with tuberculin skin testing for all individuals living with HIV [20]. In addition, *Pneumocystis* (*carinii*) *jiroveci* pneumonia prophylaxis was previously recommended in patients with CD4 >200 cells/μL combined with anergy [11]. Since DTH test methodology can assess CMI *in vivo*, DTH results can provide information about immune function in addition to the measurement of CD4 counts. DTH anergy has also been suggested as an indicator for initiating HAART in resource-limited settings [10, 11]. Although DTH results can provide prognostic information for patients, DTH testing methods (with the exception of tuberculin skin testing) are not commonly used in current clinical practice in higher income countries due to technical and resource requirements.

DTH responsiveness to recall antigens in HIV-infected patients is associated with the magnitude of the CD4 count at the time of testing. For example, one study in our military population found that 60% of treatment-naïve patients with CD4 counts less than 200 cells/μL were completely anergic, whereas 86% with >400 cells/μL were non-anergic [6]. Similarly, we observed that the median CD4 cell count for non-anergic subjects was >400 cells/μL in the current study. The median CD4 gain of 227 cells/μL post-HAART was also similar to a previously published report in our larger cohort [21]. Although DTH responsiveness is associated with the magnitude of CD4 cell count at HI, no difference in CD4 gains was observed post-HAART based on DTH status. Although DTH anergy is a predictor of disease progression in untreated HIV infection, our results do not support DTH responsiveness as a predictor of post-HAART CD4 reconstitution.

HAART has been previously shown to enhance DTH responsiveness in patients living with HIV who are anergic. In a study by Carr *et al*. [12], DTH responsiveness significantly improved over time but remained impaired for most individuals 48 weeks post-HAART. These results are in contrast to our study, in which the majority of participants showed improved DTH responsiveness. This difference may be partly explained by lower mean pre-HAART CD4 cell counts (<300 cells/μL) compared to our study (400 cells/μL), since pre-HAART CD4 cell nadir has been inversely associated with DTH responses on HAART [17, 22]. A separate study showed that improved DTH responsiveness was associated with post-HAART VL suppression [14]. In the study by Carr *et al*., 40% of participants did not achieve VL suppression on HAART, whereas our study included only VL-suppressed individuals [12]. In contrast to HIV-infected persons with suppressed VL secondary to HAART, there is an uncommon subgroup of individuals with spontaneous VL suppression in the absence of HAART termed “HIV controllers” [23]. We previously reported that DTH responsiveness was no different between HIV controllers and VL-suppressed participants on HAART when adjusting for CD4 count [15]. Thus, it appears that CD4 count at the time of DTH testing has a larger impact on DTH responsiveness than VL suppression; however, CD4 gains post-HAART are optimal in the setting of suppressed VL.

One interesting observation in this study was that DTH normalization occurred in approximately 60% of anergic participants regardless of pre-HAART CD4 cell count and prior AIDS diagnoses. A previous study showed that *in vitro* loss of T-cell function in patients living with HIV was progressive and sequential, beginning with impairments to antigen recall responses, then alloantigen responses, and ultimately mitogen-induced responses [24]. Since recall antigen responses are lost first after HIV infection, it is possible that these responses may be recovered early after initiation of effective HAART. It is important to note that HAART is not universally restorative and that a proportion of patients do not regain DTH responses, including a proportion of participants with early HIV disease and higher CD4 cell counts at the time of HI. The results of this study also demonstrate that CD4 nadir prior to HAART does not appear to blunt the ability to reverse DTH anergy and reconstitute CD4 cells after initiation of VL-suppressive HAART.

Patients in the NHS cohort have free access to healthcare and medications, which mitigates social and economic issues that can confound analyses related to race and ethnicity in other cohorts. Multiple logistic regression analyses in the current study revealed that European Americans were more likely to be anergic before HI and less likely to normalize DTH responses during HAART. Since this finding was not attributable to low CD4 counts or the presence of AIDS diagnoses, other immunologic and/or genetic aspects may have contributed to the lack of DTH responses in this group. For example, CCL3L1-CCR5 GRG status, a genetic characteristic, has been associated with differences in DTH responsiveness [25]. DTH normalization also occurred less frequently with NNRTI-based regimens, although analyses comparing HAART regimens did not differ in CD4 gains. This may be due to the small number of anergic participants initiating HAART with NNRTI-based therapy (*n*=14) compared to PI-based HAART (*n*=64). Alternatively, this finding may also be related to race because four of the five participants on NNRTI-based regimens who failed to normalize DTH responses were European Americans. Further studies are necessary to investigate the impact of racial and genetic background on DTH responsiveness and immune recovery during HAART.

In this study, the overall number of DTH responses was recorded, although detail about specific antigen responses was not available. Another potential limitation is that treatment occurred in the early HAART era. However, this was mitigated by the requirement of VL suppression for all participants included in this study. Other factors that may influence DTH responsiveness, such as vitamin deficiencies and nutritional status, were not measured, and some comparisons may have been limited by the relatively small sample size. The overall proportion of participants who were anergic at HI was lower (30%) compared to other studies, but this is likely due to relatively preserved pre-HAART CD4 counts (median 400 cells/μL) and the younger age (median 35 years) at the time of HI.

## Conclusions

DTH testing is a clinical tool that can be used to assess CMI. Although DTH responsiveness can predict HIV disease progression independent of CD4 count, pre-HAART DTH status does not appear to be predictive of CD4 gains after 24 months of VL-suppressive HAART. In participants with cutaneous anergy prior to HAART, a normalization of DTH responses occurred in approximately 60% of subjects after 24 months of suppressive HAART. In those with cutaneous anergy and advanced HIV disease, as evidenced by low CD4 cell counts and AIDS diagnoses, the capacity for both CD4 reconstitution and normalization of DTH responses during HAART appears to be similar to that of those without advanced disease.
